# RNA-binding protein Miso/CG44249 is crucial for minor splicing during oogenesis in *Drosophila*

**DOI:** 10.1261/rna.080311.124

**Published:** 2025-06

**Authors:** Yuki Taira, Li Zhu, Ryuya Fukunaga

**Affiliations:** Department of Biological Chemistry, Johns Hopkins School of Medicine, Baltimore, Maryland 21205, USA

**Keywords:** splicing, RNA-binding protein, snRNA, oogenesis, *Drosophila*

## Abstract

Pre-mRNA introns are removed by two distinct spliceosomes: the major (U2-type) spliceosome, which splices over 99.5% of introns, and the minor (U12-type) spliceosome, responsible for a rare class of introns known as minor introns. While the major spliceosome contains U1, U2, U4, U5, and U6 small nuclear RNAs (snRNAs) along with numerous associated proteins, the minor spliceosome comprises U11, U12, U4atac, U5, and U6atac snRNAs and includes specialized proteins. The function and regulation of the minor spliceosome are critical. Mutations in its specific component, RNA-binding protein RNPC3/65K, are linked to human diseases such as primary ovarian insufficiency. In this study, we identify RNA-binding protein Miso (CG44249), which shares 31% and 27% amino acid sequence identity with human RNPC3 and RBM41, respectively, as a key factor in minor splicing and oogenesis in *Drosophila*. Miso associates with U11 and U12 snRNAs in ovaries. *miso* mutant females exhibit smaller ovaries, reduced germline stem cell numbers, disrupted oogenesis, reduced fecundity, and lower fertility. In *miso* mutant ovaries, significant minor intron retention is observed, accompanied by a reduction in spliced RNAs and protein products. Our findings establish Miso as a critical factor for minor intron splicing and underscore its essential role in *Drosophila* oogenesis.

## INTRODUCTION

mRNA splicing, an essential process for generating mature mRNAs, fine-tunes gene expression and increases proteome complexity by removing intronic sequences from precursor mRNAs (pre-mRNAs). In higher eukaryotes, mRNA splicing is carried out by two distinct spliceosomes: the major (U2-type) spliceosome, which removes the majority (>99.5%) of introns, and the minor (U12-type) spliceosome, which splices a rare class of introns known as minor introns. The major spliceosome comprises U1, U2, U4, U5, and U6 small nuclear RNAs (snRNAs) and numerous associated proteins (Tholen and Galej 2022). The minor spliceosome, in contrast, consists of U11, U12, U4atac, U5, and U6atac snRNAs and includes a unique set of proteins (Ding et al. 2023). Although many proteins are shared between the major and minor spliceosomes, the minor spliceosome includes several unique proteins such as RNPC3/65K, PDCD7/59K, SNRNP48/48K, SNRNP35/35K, ZCRB1/31K, SNRNP25/25K, ZMAT5/20K, RBM41, RBM48, SCNM1, ARMC7, PPIL2, and CRIPT in humans (Will et al. 1999, 2004; Bai et al. 2021, 2024; Norppa et al. 2024). The function and regulation of the minor spliceosome are critical. Mutations in its specific components lead to developmental defects and human diseases (Padgett 2012; El Marabti et al. 2021; Norppa et al. 2025). For example, mutations in the human RNPC3/65K are linked to hypopituitarism, growth hormone deficiency, and primary ovarian insufficiency (Argente et al. 2014; Norppa et al. 2018; Verberne et al. 2020; Akin et al. 2022; Bezen et al. 2022).

**FIGURE 1.**
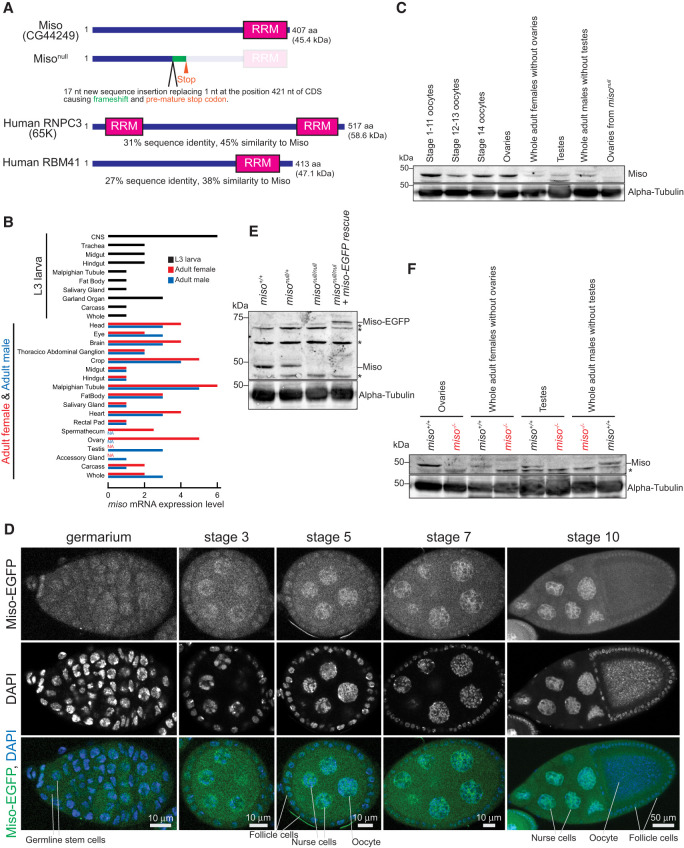
Domain structure, mutant allele, and expression of Miso. (*A*) Domain structures of *Drosophila* Miso (CG44249) protein, its null mutant allele generated in this study, and human RNPC3 (65K). (*B*) *miso* mRNA expression profile, obtained from FlyBase (FBgn0265184). (*C*) Western blot analysis of Miso protein expression. (*D*) Confocal images of germarium and egg chambers from *miso-EGFP* transgenic flies showing Miso-EGFP (green) and DAPI-stained nuclei (blue). Scale bars, 10 μm for germarium and stage 3, 5, and 7 egg chambers; 50 μm for stage 10 egg chamber. (*E*) Western blot of ovary lysates, with nonspecific bands marked by asterisk (*). (*F*) Western blot of lysates of ovaries, female carcass after ovary dissection, testes, and male carcass after testis dissection, with nonspecific bands marked by asterisk (*).

Human RNPC3/65K has two RNA-recognition motifs (RRMs) (Fig. 1A). The N-terminal half of RNPC3, which contains the N-terminal RRM, binds U11-associated PDCD7/59K protein, while the C-terminal RRM directly binds U12 snRNA by recognizing its terminal hairpin within the 3′ stem–loop (Benecke et al. 2005). Thus, RNPC3 functions as a bridge between U11-PDCD7/59K snRNP and U12 snRNA in the minor spliceosome. Human RBM41, which contains a single C-terminal RRM (Fig. 1A) and is paralogous to RNPC3, also binds U12 snRNA but not U11 snRNA (Norppa et al. 2024). RBM41 is suggested to function in the post-splicing steps of the minor spliceosome assembly/disassembly cycle (Norppa et al. 2024).

Among snRNAs, U11 and U12 snRNAs exhibit greater species divergence compared to other snRNAs in the major and minor spliceosomes (Otake et al. 2002; Schneider et al. 2004). Human and *Drosophila* U11 snRNAs are 135 and 275 nt long, respectively, and U12 snRNAs are 150 and 238 nt long, respectively, yet they retain characteristic stem–loop structures.

*Drosophila* CG44249, which has a single RRM in the C-terminal region, shares 31% sequence identity and 45% similarity with human RNPC3, and 27% sequence identity and 38% similarity with human RBM41 (Fig. 1A; Supplemental Fig. S1). The biological and molecular functions of CG44249 remain poorly understood. Thus, studying this protein may reveal both conserved and unique functions among homologous proteins and provide insights into how RNPC3 mutations are linked to diseases like hypopituitarism, growth hormone deficiency, and primary ovarian insufficiency in humans.

In this study, we examined the biological and molecular functions of CG44249. We found that CG44249 protein is highly expressed in ovaries and oocytes, and females lacking *CG44249* have smaller ovaries, fewer germline stem cells (GSCs), abnormal oocytes, lower fecundity, and decreased fertility compared to controls, showing parallels to primary ovarian insufficiency symptoms. Germline-specific transgenic expression of CG44249 restored fecundity and fertility in *CG44249* mutants. We found that minor intron splicing is impaired in *CG44249* mutant ovaries. Mechanistically, we demonstrated that CG44249 associates with U11 and U12 snRNAs in ovaries. These findings indicate that by binding U11 and U12 snRNAs, CG44249 plays a crucial role in minor splicing in ovaries, which is essential for proper oogenesis. Based on these observations, we have named *CG44249 miso* (minor splicing and oogenesis).

## RESULTS

### Miso is highly expressed in ovaries and oocytes

High-throughput mRNA expression data indicate that *miso* mRNA is expressed in various tissues in both males and females (Fig. 1B). To examine Miso protein expression, we generated a polyclonal anti-Miso antibody against a recombinant full-length Miso protein. Using this antibody in western blots, we observed high-level expression of Miso protein in ovaries and oocytes (Fig. 1C), suggesting its critical role in these tissues.

Oogenesis, the female-specific process of gametogenesis, transforms GSCs in the ovaries into mature oocytes, which accumulate large quantities of RNA essential for early embryogenesis. To determine Miso protein localization in the ovaries, we generated *miso-EGFP* transgenic flies expressing Miso with an EGFP tag at the C-terminus under the control of the endogenous *miso* promoter. Confocal imaging revealed that Miso*-*EGFP is expressed in germline cells including GSCs, cyst cells, nurse cells, and developing oocytes, as well as in somatic follicle cells, with enrichment in the nucleus (Fig. 1D).

### *miso* mutant flies

To investigate Miso function in vivo, we created a *miso* mutant allele (*miso*^*null*^) using CRISPR/Cas9, introducing a 17 nt insertion replacing a single nucleotide within the Miso coding region. This mutation causes a frameshift and a premature stop codon, resulting in a 140-amino acid (aa) N-terminal fragment of Miso followed by a 24-aa segment from frameshifted translation, lacking the C-terminal RRM (Fig. 1A). This truncated form likely lacks functionality and we were unable to detect stable protein expression, as shown below. Thus, we consider this allele a null mutation. Homozygous *miso*^*null/null*^ flies were viable, revealing that Miso is not essential for survival.

To validate the *miso*^*null*^ strain and the Miso antibody, we performed western blots using ovary lysates from *miso*^*null*^ mutant flies and the anti-Miso antibody. As expected, full-length Miso protein was detected in the ovaries of wild-type (*miso*^+/+^) and heterozygous (*miso*^*null/*+^) controls, but not in homozygous mutants (*miso*^*null/null*^) (Fig. 1C,E). No smaller protein corresponding to the truncated Miso fragment was detected in either *miso*^*null/*+^ or *miso*^*null/null*^ ovaries, suggesting instability or no-expression of this fragment. We also performed western blots using recombinant Miso protein expressed in and purified from *Escherichia coli* as a control and it exhibited similar electrophoresis mobility to the protein detected in *miso*^+/+^ ovaries, further confirming that the detected protein corresponds to Miso protein (Supplemental Fig. S2). We also performed western blots using female carcasses after removing ovaries, male testes, and male carcasses after removing testes, and confirmed that Miso expression is absent in these *miso*^*null/null*^ samples (Fig. 1F).

### Miso is important for female fecundity and fertility

**FIGURE 2.**
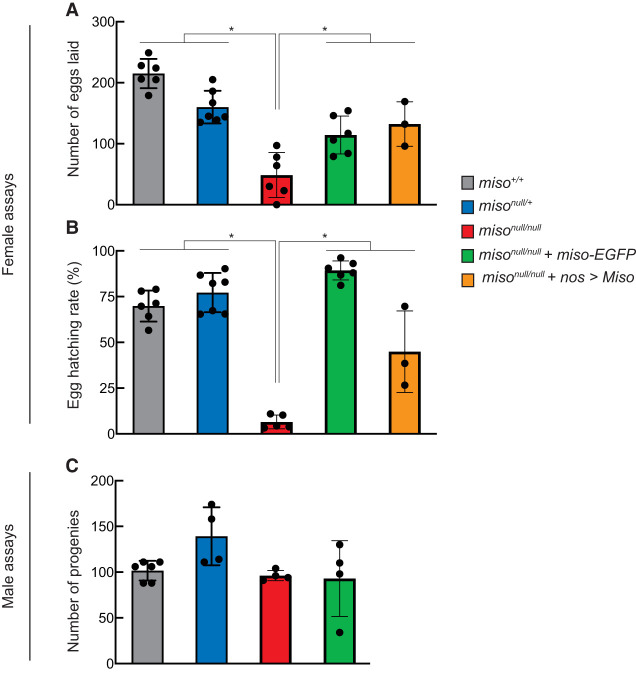
Reduced fecundity and fertility in *miso* mutant females. (*A* and *B*) Female fertility assays. (*A*) Egg counts from test females mated with wild-type (Oregon-R) males. (*B*) Egg hatching rates. Mean ± SD (*n* = 6, 7, 6, 6, and 3, for *miso*^+/+^, *miso*^*null/*+^, *miso*^*null/null*^, *miso*^*null/null*^ + *miso-EGFP*, and *miso*^*null/null*^ + *nos > Miso*, respectively). *P*-value <0.05 (Student's *t*-test, unpaired, two-tailed) is indicated by *. (*C*) Male fertility assays showing progeny counts from test males crossed with wild-type (Oregon-R) virgin females. Mean ± SD (*n* = 6 for *miso*^+/+^ and 4 for *miso*^*null/*+^, *miso*^*null/null*^, and *miso*^*null/null*^ + *miso-EGFP*).

Given the high expression of Miso in ovaries (Fig. 1C), we hypothesized that it plays a critical role in female fertility. To test this, we performed female fertility assays by mating virgin females of controls (*miso*^+/+^ and *miso*^*null/*+^) and *miso*^*null/null*^ with wild-type (Oregon-R) males. The number of eggs laid by *miso*^*null/null*^ females was significantly decreased compared to controls (Fig. 2A). Furthermore, the hatching rate of the eggs laid by *miso*^*null/null*^ females was markedly lower than those of controls (Fig. 2B). To confirm these defects were due to *miso* loss, we generated Miso rescue flies expressing Miso-EGFP in *miso*^*null/null*^ background (*miso*^*null/null*^; *miso-EGFP*. Hereafter “Miso-EGFP rescue”). Western blot analysis confirmed that these flies express Miso-EGFP at levels comparable to endogenous Miso in control flies, without expressing endogenous Miso (Fig. 1E). Both fecundity and fertility of the Miso-EGFP rescue flies were significantly higher compared to *miso*^*null/null*^ (Fig. 2A,B). These results demonstrated that Miso is important for female fecundity and fertility.

To determine whether germline-specific expression of Miso could restore female fecundity and fertility, we used the GAL4-UAS system to express transgenic Miso (*UASp-3×HA-HRV3Ccleavagesite-3×FLAG-Miso*, hereafter “*UASp-HHF-Miso*”) in *miso*^*null/null*^ with a germline-specific driver, *nanos* (*nos*)*-GAL4* (*miso*^*null*^, *nos-GAL4*/*miso*^*null*^; *UASp-HHF-Miso/*+). We confirmed transgenic protein expression of HHF-Miso in ovaries (Supplemental Fig. S3). The number of eggs laid and the hatching rate of this germline-specific Miso rescue flies were significantly higher than those of *miso*^*null/null*^, indicating that germline expression of Miso is sufficient to restore female fecundity and fertility (Fig. 2A,B).

We also conducted male fertility assays by mating virgin males with wild-type (Oregon-R) virgin females. The number of progenies from *miso*^*null/null*^ males was not significantly different from those of controls and Miso-EGFP rescue males (Fig. 2C), demonstrating that Miso is dispensable for male fertility. We conclude that *miso* is important for female fecundity and fertility but not for male fertility, and that germline-specific Miso expression is sufficient to rescue female fecundity and fertility.

### Miso is important for ovary size and oocyte quality

**FIGURE 3.**
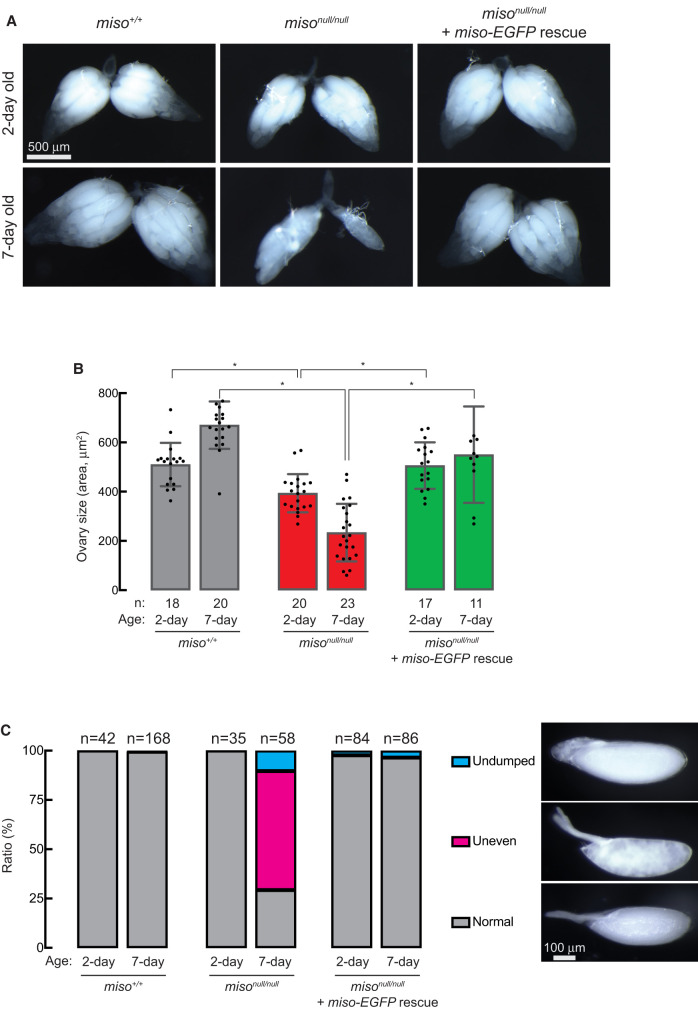
Reduced ovary size and abnormal stage 14 oocytes in *miso* mutant females. (*A*) Stereomicroscope images of dissected ovaries. Scale bar, 500 μm. (*B*) Quantification of ovary size (area) from dissected samples. Mean ± SD. *P*-value < 0.05 (Student's *t*-test, unpaired, two-tailed) is indicated by *. (*C*) Percentage of stage 14 (mature) oocytes with normal, uneven, and undumped phenotypes. Representative images of these phenotypes are displayed. Scale bar, 100 μm.

To explore how Miso affects female fecundity and fertility, we assessed ovary size and oocyte quality. Ovaries from 2- and 7-day-old *miso*^*null/null*^ were notably smaller than those of age-matched control (*miso*^+/+^) and Miso-EGFP rescue flies, with differences increasing with age (Fig. 3A,B).

We examined the morphology of stage 14 (mature) oocytes in the ovaries. Nearly all mature oocytes from 2-day-old *miso*^*null/null*^ females displayed normal morphology, similar to controls and Miso-EGFP rescue (Fig. 3C). However, in 7-day-old *miso*^*null/null*^ females, ∼60% of oocytes had uneven cytoplasmic contents (“uneven” phenotype), while 10% retained nurse cells at the anterior tip (“undumped” phenotype), compared to nearly all normal oocytes in controls and Miso-EGFP rescue flies. These results suggest that Miso is critical for ovary size and mature oocyte quality.

### Miso is important for maintaining GSC numbers

**FIGURE 4.**
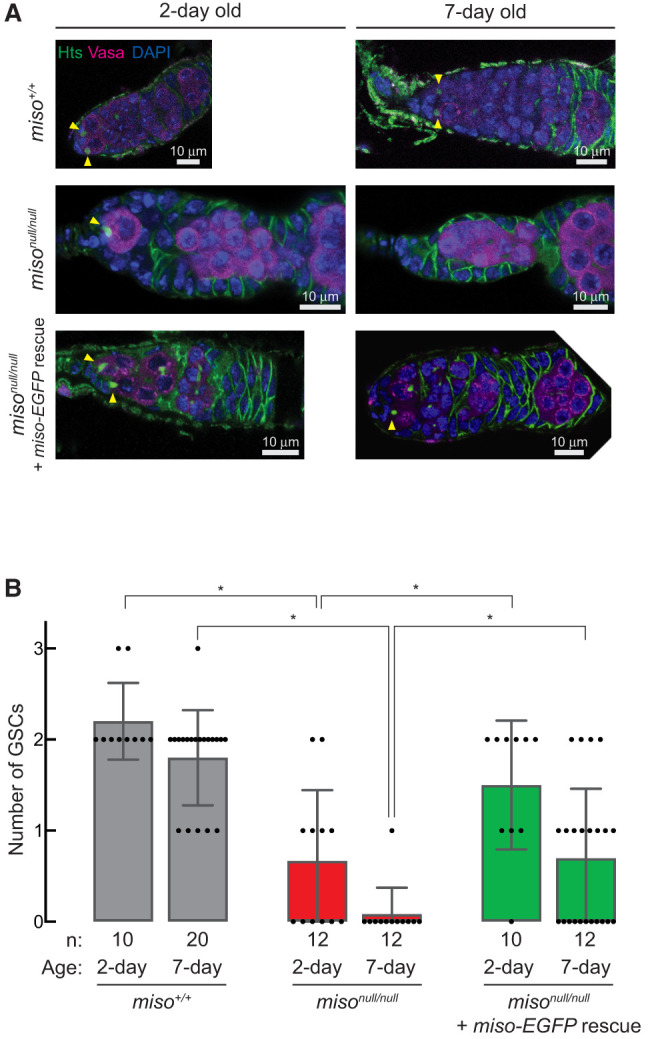
Reduced GSC numbers in *miso* mutant females. (*A*) Confocal images of germaria stained with anti-Hts (spectrosome) and anti-Vasa (germline cells) antibodies. Yellow triangles indicate GSCs, which have round spectrosome contacting cap cells at the anterior tip. Scale bar, 10 μm. (*B*) Quantification of GSCs per germarium. Mean ± SD. *P*-value < 0.05 (Student's *t*-test, unpaired, two-tailed) is indicated by *.

Next, we examined whether Miso is essential for maintaining GSC numbers. We performed immunostaining of ovaries using anti-Hts antibody to stain spectrosome and anti-Vasa antibody to stain germline cells and counted the number of GSCs, which have a round spectrosome contacting the cap cells (Fig. 4A). In 2-day-old control flies (*miso*^+/+^), we observed 2.2 ± 0.4 GSCs per germaria (Fig. 4). In contrast, 2-day-old *miso*^*null/null*^ had only 0.67 ± 0.75 GSCs per germaria (*P*-value [vs. *miso*^+/+^] = 1.8 × 10^−5^). The GSC count in 2-day-old Miso-EGFP rescue flies was rescued to 1.5 ± 0.7 GSCs per germaria (*P*-value [vs. *miso*^*null/null*^] = 0.017). The disparity in GSC counts was even greater in 7-day-old flies. The numbers of GSCs in the 7-day-old *miso*^+/+^, *miso*^*null/null*^, Miso-EGFP rescue flies were 1.8 ± 0.5, 0.08 ± 0.27, and 0.70 ± 0.75, respectively (*P-*value [*miso*^+/+^ vs. *miso*^*null/null*^] = 1.8 × 10^−11^; *P*-value [*miso*^*null/null*^ vs. Miso-EGFP rescue] = 0.012). These results reveal that Miso is important for maintaining GSC numbers.

### Miso associates with U11 and U12 snRNAs in ovaries

**FIGURE 5.**
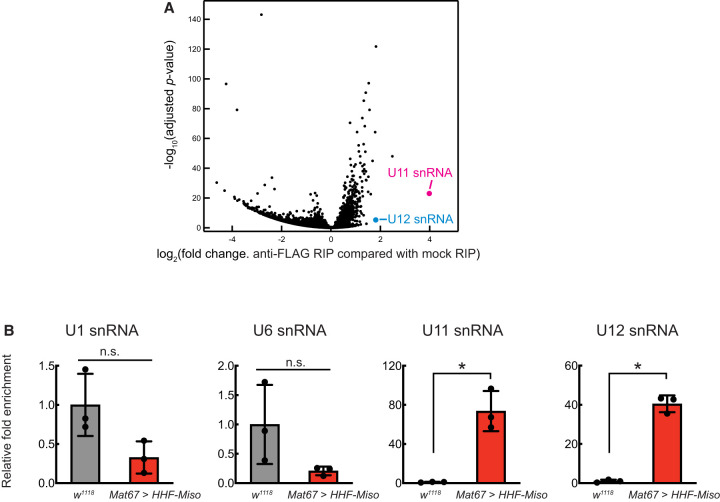
Miso associates with U11 and U12 snRNAs in ovaries. (*A*) Volcano plots from RIP-seq of anti-FLAG immunoprecipitates from *Mat67* > *UASp-HHF-Miso* ovary lysates compared to IgG controls. Mean values from three biological replicates are shown. (*B*) RT-qPCR quantification of U1, U6, U11, and U12 snRNAs in anti-HA immunoprecipitates from *Mat67* > *UASp-HHF-Miso* ovaries compared to control *w*^*1118*^ samples, normalized to *act5c*, *gapdh*, and *rp49* mRNAs. Mean ± SD (*n* = 3). *P*-value < 0.05 (Student's *t*-test, unpaired, two-tailed) is indicated by *.

To identify RNAs associated with Miso, we performed RNA immunoprecipitation (RIP) with anti-FLAG beads, followed by high-throughput sequencing (RIP-seq) using ovary lysates from flies expressing *UASp-HHF-Miso* driven by the germline-specific *Mat67-GAL4* driver (*mat67* > *HHF-Miso*). Mock immunoprecipitation with mouse IgG served as a negative control. U11 snRNA and U12 snRNA exhibited the highest and fourth highest fold enrichment, respectively, in the anti-FLAG Miso immunoprecipitates compared to the negative control (Fig. 5A; Supplemental File S1). To validate this, we conducted RIP from *mat67* > *HHF-Miso* ovary lysates using anti-HA beads followed by reverse-transcription and quantitative PCR (RT-qPCR) with anti-HA RIP from *w*^*1118*^ fly ovaries (lacking transgenic HHF-Miso) as a negative control. This analysis again revealed significant enrichment of U11 and U12 snRNAs in anti-HA Miso immunoprecipitates (Fig. 5B). In contrast, the major spliceosomal U1 and U6 snRNAs were not enriched, indicating that Miso associates specifically with U11 and U12 snRNAs in ovaries.

### Miso is important for minor splicing in ovaries

**FIGURE 6.**
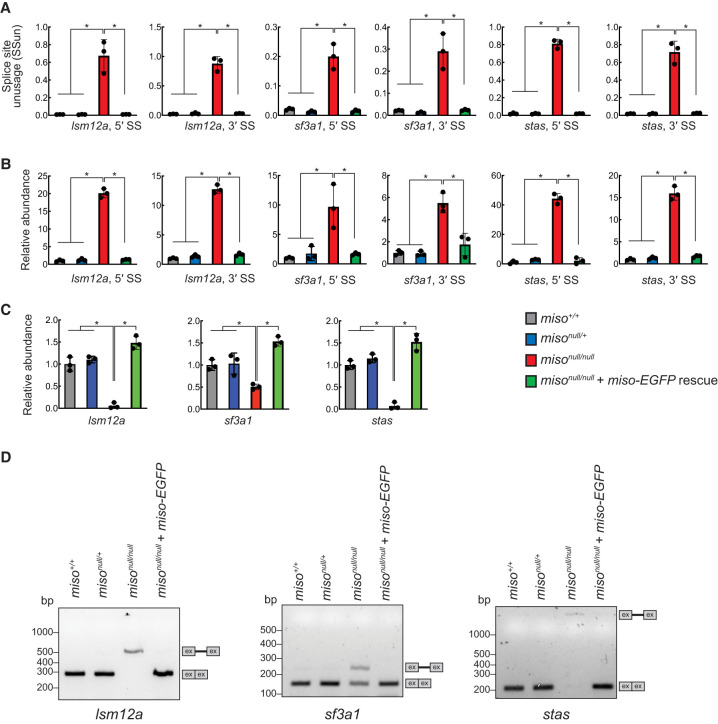
Minor intron retention and reduced spliced mRNAs from MIGs in *miso* mutant ovaries. (*A*) SSun of 5′ and 3′ splice sites of minor introns, as determined by RNA-seq of ovary RNAs. Mean ± SD (*n* = 3). *P*-value < 0.05 (Student's *t*-test, unpaired, two-tailed) is indicated by *. (*B*) RT-qPCR quantification of RNAs with retained minor introns at 5′ and 3′ splice sites, normalized to *act5c* mRNA. Mean ± SD (*n* = 3). *P*-value < 0.05 (Student's *t*-test, unpaired, two-tailed) is indicated by *. (*C*) RT-qPCR quantification of spliced mRNAs from MIGs and *gapdh2* in ovaries, normalized to *act5c* mRNA. Mean ± SD (*n* = 3). *P*-value < 0.05 (Student's *t*-test, unpaired, two-tailed) is indicated by *. (*D*) Agarose gel electrophoresis of RT-PCR products from ovary RNAs, stained with SYBR Safe. Bands representing spliced and unspliced RNAs are indicated. Results for *lsm12a*, *sf3a1*, and *stas* are shown as representative. The results for the other MIGs and *gapdh* are shown in Supplemental Figures S4, S6, and S7.

To investigate Miso's role in minor splicing, we conducted RNA-seq on ovary RNAs from control (*miso*^+/+^ and *miso*^*null/*+^), *miso*^*null/null*^, and Miso-EGFP rescue flies. Using these data, we assessed transcriptome-wide splicing by calculating splice site unusage (SSun) values, defined as the ratio of exonic boundary reads to intronic boundary reads (Li et al. 2020). *Drosophila melanogaster* contains 19 minor introns (Lin et al. 2010); we determined SSun values for 14 of these in our ovary RNA-seq data. In *miso*^*null/null*^, SSun values for the 5′ and 3′ splice sites of these 14 minor introns were significantly elevated compared to other genotypes (Fig. 6A; Supplemental Fig. S4A; Supplemental File S2. The results for *lsm12a*, *sf3a1*, and *stas* are shown in Fig. 6 as representative, while the results for the other minor intron-containing genes (MIGs) are shown in Supplemental Fig. S4; Supplemental File S2). SSun values of a *gapdh2* major intron showed no difference (Supplemental Fig. S4B). While we identified 33 major introns whose both 5′ and 3′ splice sites have significantly higher SSun values in *miso*^*null/null*^ than in *miso*^+/+^, *miso*^*null/*+^, and Miso-EGFP rescue flies, the difference was much smaller compared with those of minor introns (Supplemental Fig. S5; Supplemental File S3), showing that *miso* mutation affects predominantly minor splicing but not major splicing.

We further validated these findings by RT-qPCR to quantify RNAs with retained minor introns. RNAs containing retained minor introns produced from the 14 MIGs showed higher abundance in *miso*^*null/null*^ relative to other genotypes (Fig. 6B; Supplemental Fig. S4C). No changes were observed in the abundance of *gapdh2* RNAs with retained major introns (Supplemental Fig. S4D).

We also quantified spliced RNAs lacking introns using RT-qPCR with primer sets spanning exon–exon junctions, designed to target spliced RNA only. Most of the 14 MIGs showed significantly lower spliced RNA abundance in *miso*^*null/null*^ ovaries compared to other genotypes, whereas the spliced *gapdh2* RNA abundance was similar across genotypes (Fig. 6C; Supplemental Fig. S6).

To confirm minor intron retention, we performed RT-PCR on ovary RNAs, followed by agarose gel electrophoresis using primers targeting exons flanking the minor introns. In *miso*^*null/null*^, almost all MIGs showed unspliced amplicons, with reduced intensity of the spliced amplicons, compared to other genotypes (Fig. 6D; Supplemental Fig. S7). The *gapdh2* major intron was efficiently spliced in all genotypes. Together, these results revealed that minor splicing is impaired in *miso*^*null/null*^ ovaries.

Previous studies showed that minor spliceosome inhibition can cause alternative splicing that uses cryptic splice sites in MIGs (Li et al. 2020; Olthof et al. 2021; Augspach et al. 2023). We examined if such aberrant splicing using cryptic splice sites in MIGs is increased in *miso*^*null/null*^. Indeed, we found that cryptic splicing is increased in Tsp97E. Cryptic splicing using the normal 5′ splice site of the Tsp97E major intron 1 (downstream from exon 1) and one of two cryptic 3′ splice sites within exon 3 (downstream from the Tsp97E minor intron) are significantly increased and the normal splicing of the major intron 1 (intron between exon 1 and exon 2) is significantly decreased in *miso*^*null/null*^ ovaries compared with *miso*^+/+^, *miso*^*null/*+^, and Miso-EGFP rescue flies (Supplemental Fig. S8). Thus, our data support that minor splicing inhibition can cause alternative splicing that uses cryptic splice sites in MIGs.

**FIGURE 7.**
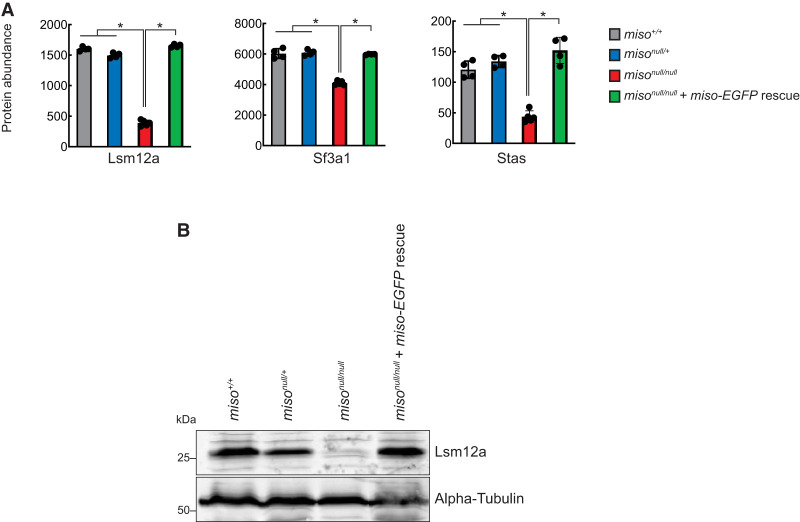
Reduced protein levels of MIGs in *miso* mutant ovaries. (*A*) Protein levels of MIGs in ovaries, quantified by TMT mass spectrometry. Mean ± SD (*n* = 4 for *miso*^+/+^, *miso*^*null/*+^, and *miso*^*null/null*^ + *miso-EGFP*; 5 for *miso*^*null/null*^). *P*-value < 0.001 (Student's *t*-test, unpaired, two-tailed) is indicated by *. Results for Lsm12a, Sf3a1, and Stas are shown as representative. The results for the other MIGs and Gapdh2 are shown in Supplemental Figure S9. (*B*) Western blot of ovary lysates for Lsm12a.

We next quantified protein abundances in ovaries from control (*miso*^+/+^ and *miso*^*null/*+^), *miso*^*null/null*^, and Miso-EGFP rescue flies using tandem mass tag (TMT) mass spectrometry. Proteins from 12 MIGs were detected, and eight (CG11839, Lsm12a, Naa60, Nhe3, Phb2, Sf3a1, Stas, Tsp97E) showed significantly lower levels in *miso*^*null/null*^ compared to other genotypes (Fig. 7A; Supplemental Fig. S9). Gapdh2 protein levels remained unchanged across genotypes. To validate these findings, we performed western blot for Lsm12a and confirmed that its level is decreased in *miso*^*null/null*^ ovaries, consistent with the mass-spec results (Fig. 7B).

Taken together, these results demonstrate that in *miso*^*null/null*^ ovaries, minor introns are retained in transcripts, leading to decreased levels of spliced RNAs and proteins, indicating that Miso is crucial for minor splicing.

### Differential expression analysis of mRNAs and proteins in miso mutant ovaries

We performed differential expression analysis using our RNA-seq data and TMT mass-spec data. RNA-seq data analysis identified 441 differentially expressed genes in *miso*^*null/null*^ ovaries, including 51 significantly downregulated and 390 upregulated genes (adjusted *P*-value <0.05 and log_2_(fold-change) < −0.29 or >0.29 in *miso*^*null/null*^ vs *miso*^+/+^, *miso*^*null/*+^ and Miso-EGFP rescue flies. Supplemental File S4). Mass-spec data analysis identified 169 differentially expressed proteins in *miso*^*null/null*^ ovaries (28 downregulated and 141 upregulated, adjusted *P*-value <0.05 and fold-change <0.7 or >1.43 in *miso*^*null/null*^ vs *miso*^+/+^, *miso*^*null/*+^, and Miso-EGFP rescue flies. Supplemental File S5). Using these lists of differentially expressed genes and proteins, we performed gene ontology (GO) term enrichment analyses, which indicated dysregulation at both RNA and protein levels of some pathways such as actomyosin structure organization, muscle system process, cellular component assembly involved in morphogenesis, myofibril assembly, actin cytoskeleton organization, and actin filament-based process (Supplemental Tables S1, S2). Actomyosin structure and actin cytoskeleton organization and processes are crucial for oogenesis (Robinson and Cooley 1997; Wang and Riechmann 2007; Leibfried et al. 2013; Quinlan 2013; Drechsler et al. 2017; Sokolova et al. 2018; Li et al. 2024b; Wood et al. 2024). Their dysregulation may underlie female reproductive defects in *miso* mutants. However, no MIGs are annotated to the pathways described above and it remains unknown how *miso* mutation caused observed differential expression of the responsible RNAs and proteins for the pathways.

### Miso is not important for abundance of U11 and U12 snRNAs

**FIGURE 8.**
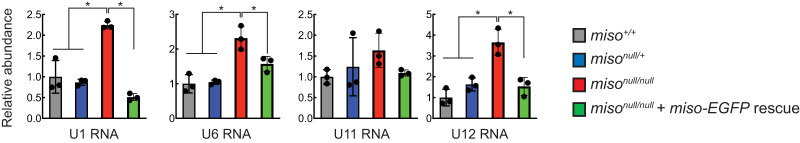
Increased U1, U6, and U12 snRNAs in *miso* mutant ovaries. RT-qPCR quantification of U1, U6, U11, and U12 snRNAs in ovaries normalized to *act5c* mRNA. Mean ± SD (*n* = 3). *P*-value < 0.05 (Student's *t*-test, unpaired, two-tailed) is indicated by *.

We examined whether U11 and U12 snRNA levels were affected in *miso*^*null/null*^, hypothesizing that Miso might stabilize these RNAs. RT-qPCR analysis revealed no reduction in U11 or U12 snRNA levels in *miso*^*null/null*^ compared to other genotypes; instead, U1, U6, and U12 snRNA levels were slightly increased in *miso*^*null/null*^ (Fig. 8). We speculate that this suggests a possible feedback mechanism to compensate for diminished minor splicing activity in Miso's absence.

## DISCUSSION

In this study, we showed that Miso is highly expressed in ovaries and oocytes with nuclear enrichment (Fig. 1) and associates with U11 and U12 snRNAs in ovaries (Fig. 5). We demonstrated that minor splicing is impaired in *miso*^*null/null*^ ovaries (Figs. 6, 7), supporting Miso's role as a critical component of the minor spliceosome.

Miso shares a higher sequence similarity with human RNPC3 than with RBM41, although both Miso and RBM41 possess a single C-terminal RRM, whereas RNPC3 contains two RRMs (Fig. 1A; Supplemental Fig. S1). RNPC3 directly binds U12 snRNA and interacts with PDCD7/59K to engage U11 snRNA (Benecke et al. 2005; Bai et al. 2024). In contrast, RBM41 associates with U12 snRNA but does not interact with U11 snRNA (Norppa et al. 2024). Our finding that Miso associates with both U11 and U12 snRNAs in ovaries (Fig. 5) suggests that Miso functions as a bridging factor within the minor spliceosome, similar to RNPC3's proposed role (Benecke et al. 2005). *Drosophila* U11 and U12 snRNAs are notably longer (275 and 238 nt long, respectively) than their human counterparts (135 and 150 nt long), which may impact minor spliceosome assembly and function across species. Future studies are needed to elucidate how Miso associates with U11 and U12 snRNAs and contributes to minor splicing reactions.

While RNPC3 knockout mice are embryonically lethal (Doggett et al. 2018) and loss of U12 or U6atac snRNA results in embryonic, larval, or pupal lethality in *Drosophila* (Otake et al. 2002; Li et al. 2020), *miso*^*null*^ mutant flies are viable and thus *miso* is not essential for organismal viability. However, *miso*^*null*^ mutants exhibit significant female reproductive defects, including smaller ovaries (Fig. 3), fewer GSCs (Fig. 4), abnormal oocytes (Fig. 3), and reduced fecundity and fertility (Fig. 2A,B), without male reproductive change (Fig. 2C). Notably, all defects were rescued by the *miso-EGFP* transgene, and fecundity and fertility were restored by germline-specific transgenic Miso expression (Fig. 2A,B), indicating that the primary defects arise from the loss of Miso function in the germline rather than other tissues, such as the brain (Li et al. 2020, 2024a).

Human RNPC3 mutations have been associated with hypopituitarism, growth hormone deficiency, and primary ovarian insufficiency—conditions that involve early ovarian failure and infertility (Argente et al. 2014; Norppa et al. 2018; Verberne et al. 2020; Akin et al. 2022; Bezen et al. 2022). Our findings that *miso* mutant females have impaired ovaries with reduced fecundity and fertility mirrors these human phenotypes, suggesting a conserved role for RNPC3/Miso in female reproductive health. Given that germline-specific transgenic expression of Miso rescued fecundity and fertility in *miso*^*null*^ mutant females, restoring RNPC3 function in human germ cells may represent a potential therapeutic strategy.

The female-specific reproductive defects observed in *miso*^*null*^ mutants, without impact on viability or male fertility, are intriguing. This suggests that minor splicing may be more dependent on Miso in the ovaries and/or that the functions of MIGs are more critical in this tissue. However, functional annotation of MIGs (Supplemental Table S3) revealed no apparent direct link to the female reproductive system. Future studies exploring whether expressing select MIGs in ovaries can restore normal female reproductive function would be valuable to understand the molecular underpinnings linking among *miso* mutation, minor splicing defects, and female reproductive defects.

In summary, our studies demonstrate that Miso associates with U11 and U12 snRNAs, playing a critical role in minor splicing and oogenesis. Further investigations are needed to elucidate the detailed molecular functions and mechanism of Miso and how female reproductive defects are caused in *miso* mutants.

## MATERIALS AND METHODS

### Fly strains

The *miso*^*null*^ strain was generated by introducing indels within the Miso coding region using the CRISPR/Cas9 genome editing system, as described previously (Zhu et al. 2018a, b, 2019b; Zhu and Fukunaga 2021). For the transgenic *miso*-*EGFP* strain, a fragment containing Miso coding sequence, along with ∼2 kbp upstream and ∼0.8 kbp downstream genomic sequences, was cloned. The EGFP gene was fused in-frame to the C-terminus of the Miso coding sequence and inserted into a pattB plasmid vector. For the transgenic *UASp-3×HA-HRV3Ccleavagesite-3×FLAG-Miso* (*UASp-HHF-Miso*) strain, Miso coding sequence was tagged with N-terminal 3×HA-HRV3Ccleavagesite-3×FLAG and inserted into a pUASPattB plasmid vector (Takeo et al. 2012). The *miso*-*EGFP* plasmid was integrated into the 86F8 site of the fly genome using the RRID:BDSC_24749 fly strain and the PhiC31 system, and the *UASp-HHF-Miso* plasmid was integrated at the 68A4 site using the attP2 fly strain.

### Fertility assay

Fertility assays were performed as described previously (Fukunaga et al. 2012; Zhu et al. 2018b, 2019a; Liao et al. 2019; Zhu and Fukunaga 2021). Briefly, for female fertility assays, five test virgin females were mated with five wild-type (Oregon-R) males in vials with wet yeast paste for 1 day, then transferred to cages containing a 6 cm grape juice agar plate with wet yeast paste. After 1 day, the number of eggs laid on the plates was counted. After incubation of the plates at 25°C for an additional day, hatched eggs were counted. At least three cages were tested per genotype.

For male fertility assays, a single test male was mated with five wild-type (Oregon-R) virgin females in vials for 3 days. Then, females were transferred to new vials every 2 days over four vials. After 2 days in the final vials, females were removed. Total progeny from these four vials were counted. At least four males per genotype were tested.

### Miso antibodies

Recombinant full-length Miso protein, tagged with N-terminal 6×His-MBP-HRV3Ccleavagesite, was expressed in *E. coli* using a modified pET vector (Fukunaga and Doudna 2009). The protein was purified using Ni-sepharose (Cytiva), followed by cleavage of the 6×His-MBP tag with HRV3C protease. Further purification was performed using a HiTrap SP HP (Cytiva) column. Polyclonal anti-Miso sera were generated in rabbits using this antigen (Pocono Rabbit Farm & Laboratory, Inc.). Anti-Miso antibodies were affinity-purified using 6×His-MBP-Miso recombinant protein and Affigel-10 (Bio-Rad), following the manufacturer's instructions.

### Western blot

Recombinant full-length Miso protein, tagged with N-terminal 6×His-HRV3Ccleavagesite, was expressed in *E. coli* using a modified pColdI (Takara) (Fukunaga and Doudna 2009). After Ni-sepharose purification and HRV3C protease cleavage of the tag, the protein was further purified by passing through Ni-sepharose.

Western blot was performed as previously described (Kandasamy et al. 2017; Zhu et al. 2018b; Liao et al. 2019; Zhu and Fukunaga 2021). Hand-dissected tissues were homogenized in RIPA buffer (50 mM Tris-HCl [pH 7.4], 150 mM NaCl, 1% [v/v] IGEPAL CA-630, 0.1% [w/v] sodium dodecyl sulfate (SDS), 0.5% [w/v] sodium deoxycholate, 1 mM ethylenediaminetetraacetic acid [EDTA], 5 mM dithiothreitol, and 0.5 mM phenylmethylsulfonyl fluoride [PMSF]). After centrifugation at 21,000*g* for 10 min at 4°C, supernatant protein concentrations were measured using the Pierce BCA Protein Assay Kit (Thermo Fisher Scientific). Primary antibodies used were rabbit anti-Miso (1:5000, generated in this study), mouse anti-α-Tubulin [12G10] (1:1000, DSHB, AB_1157911), and rabbit anti-Lsm12a (1:2000, a kind gift from Dr. Lim [Lee et al. 2017]). Secondary antibodies were IRDye 800CW goat anti-rabbit IgG, IRDye 680RD goat anti-mouse IgG, and IRDye 680RD goat anti-rabbit (1:10,000, Li-Cor). Membranes were scanned using the Li-Cor Odyssey CLx Imaging System.

### Immunostaining

Ovaries and oocytes from yeast-fed females were dissected in 1× *Drosophila* ringer (13 mM NaCl, 4.7 mM KCl, 1.9 mM CaCl_2_) at room temperature and were imaged using Leica M125 stereomicrocsope (Zhu et al. 2019c). For confocal imaging, ovaries were fixed in a buffer containing 4% formaldehyde in PBX (0.1% Triton X-100 in 1× PBS [137 mM NaCl, 2.7 mM KCl, 10 mM Na_2_HPO_4_, 1.8 mM KH_2_PO_4_, pH 7.4]), rinsed three times with PBX, and then incubated in a blocking buffer (2% donkey serum and 3% BSA [w/v] in PBX) for 1 h at room temperature. Ovaries were then incubated overnight with primary antibodies in blocking buffer at 4°C. After three PBX rinses, secondary antibodies were applied for 2 h at room temperature. After three PBX rinses, samples were mounted in VECTASHIELD PLUS antifade mounting medium with DAPI (H-2000, Vector Laboratories). Confocal images were acquired on a Zeiss LSM700 confocal microscope at the Johns Hopkins University School of Medicine Microscope Facility. Primary antibodies used were mouse anti-HTS (1B1) (DSHB, AB_528070, 1:100) and rat anti-Vasa (DSHB, AB_760351, 1:100). Secondary antibodies were Alexa Fluor 488 Donkey anti-Mouse Igg (Thermo Fisher Scientific, A21202, 1:1000), Alexa Fluor 594 Donkey anti-Rat Igg (Thermo Fisher Scientific, A21209, 1:1000), and Alexa Fluor 594 Donkey anti-Mouse Igg (Thermo Fisher Scientific, A21203, 1:1000).

### High-throughput RNA-sequencing

RNA libraries from ovaries were prepared, sequenced on HiSeq 2500 (Illumina), and analyzed, as previously described (Zhu et al. 2018b, 2019a, b; Zhu and Fukunaga 2021). SSun, calculated as the ratio of 40 nt exonic boundary reads flanking each splice site to 40 nt intronic boundary reads, was determined as previously described (Li et al. 2020). Dysregulation of alternative splicing including cryptic splicing was analyzed using LeafCutter (Li et al. 2018). Anti-FLAG RIP was conducted on ovary lysates from *Mat67* > *UASp-HHF-Miso* flies using the Magna RIP Kit (Millipore Sigma) with mock RIP using mouse IgG as a control. Input and RIP RNAs were sequenced on DNBSEQ-G400 at Beijing Genomics Institute. SRA accession number for these data sets is PRJNA1165777. GO term enrichment analysis was performed using GOrilla (Eden et al. 2009).

### RT-qPCR and RT-PCR

Total RNA was purified from ovaries using miRVana (Thermo Fisher Scientific) or TRIsol-LS (Thermo Fisher Scientific). For RIP samples, after ovary lysates were incubated with Pierce anti-HA magnetic beads (Thermo Fisher Scientific), protein–RNA complexes were eluted by HRV3C protease cleavage, and RIP RNAs were purified using TRIsol-LS. RNAs were treated with Turbo DNase (Thermo Fisher Scientific) and were reverse-transcribed using random hexamer primers and AMV Reverse Transcriptase (NEB) or LunaScript RT SuperMix (NEB). qPCR was performed using SsoAdvanced Universal SYBR Green Supermix on CFX96 (Bio-Rad). PCR was carried out using GoTaq Green Master Mix (Promega) followed by electrophoresis on agarose gels containing SYBR Safe (Thermo Fisher Scientific) in TAE buffer (40 mM Tris, 20 mM acetic acid, and 1 mM EDTA). Primer sequences are listed in Supplemental Table S4.

### Mass spectrometry

Ovary homogenates, prepared in RIPA buffer at 0.5 mg/mL and assessed by SDS-PAGE with Coomassie staining, were flash-frozen in liquid nitrogen and stored at −80°C. TMT mass-spec was performed as described previously (Zhu and Fukunaga 2021). GO term enrichment analysis was performed using GOrilla (Eden et al. 2009).

## SUPPLEMENTAL MATERIAL

Supplemental material is available for this article.

## Figures and Tables

**Yuki Taira:**
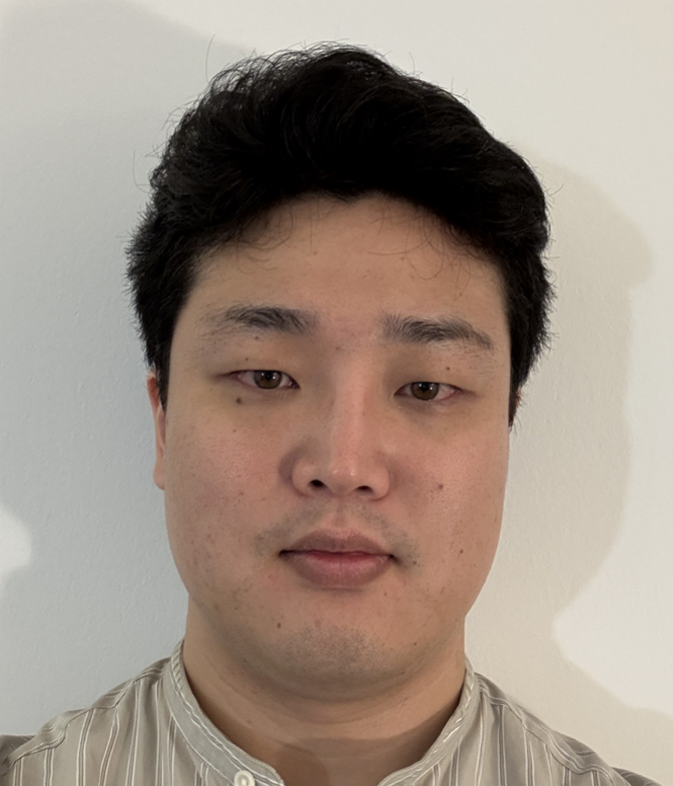


## MEET THE FIRST AUTHOR

***Meet the First Author(s)* is an editorial feature within *RNA*, in which the first author(s) of research-based papers in each issue have the opportunity to introduce themselves and their work to readers of *RNA* and the RNA research community. Yuki Taira is the first author of this paper, “RNA-binding protein Miso/CG44249 is crucial for minor splicing during oogenesis in *Drosophila.*” Yuki is a postdoctoral fellow in the Fukunaga Laboratory at Johns Hopkins University School of Medicine. His research focus is identifying RNA-binding proteins important for gametogenesis and analyzing their molecular functions.**

**What are the major results described in your paper and how do they impact this branch of the field?**

The major results of our study demonstrate that the RNA-binding protein *Miso/CG44249* associates with U11 and U12 snRNAs, ensuring female fertility. *Miso* shares amino acid sequence homology with human RNPC3/65K and RBM41, which are components of the minor spliceosome. Loss of *miso* leads to a reduction in both the number of eggs laid and the hatching rate, accompanied by a decrease in GSCs and defects in mature oocytes. *Miso* interacts with both U11 and U12 snRNAs in the ovary, *miso* mutants exhibit significant retention of minor introns, resulting in reduced levels of spliced mRNAs and protein products. These findings indicate that *Miso*, through its interaction with U11 and U12 snRNAs, plays a crucial role in minor intron splicing in the ovary, which is essential for proper oogenesis in *Drosophila*. Given that both human RNPC3/65K and *Drosophila* Miso are linked to female fertility, restoring RNPC3 function in human germ cells may hold therapeutic potential.

**What led you to study RNA or this aspect of RNA science?**

During my PhD studies, I focused on a micropeptide encoded by a small open reading frame gene, whose transcript had previously been considered a non-protein-coding RNA. This experience sparked my interest in RNA regulation and the complexity of RNA biology. After earning my PhD, I was given a great opportunity by Dr. Fukunaga to further explore this field. I became particularly fascinated by how RNA-binding proteins regulate gene expression at the posttranscriptional level, providing fine-tuned control over protein output. I am especially interested in how such precise regulation is achieved and its biological significance.

Through my research in the Fukunaga lab, I have greatly expanded both my knowledge and experimental skills and developed the ability to design studies from multiple perspectives. This has deepened my commitment to understanding RNA-mediated regulation and its role in developmental and reproductive processes.

**During the course of these experiments, were there any surprising results or particular difficulties that altered your thinking and subsequent focus?**

One surprising result was that while *U12* snRNA mutants are lethal at the pupal stage, indicating that minor splicing is essential for viability and development, *miso* mutants survive to adulthood. Based on the critical role of minor splicing, we initially expected that *Miso*, which is a homolog of a minor spliceosome component 65K, would also result in lethality. The fact that *miso* mutants were viable challenged our assumptions and prompted us to consider that some minor spliceosome components might have tissue-specific roles or varying levels of importance. Although unexpected, this outcome turned out to be an advantage, as it enabled us to analyze *Miso* function specifically in the ovary and oogenesis. It also reminded us that even within well-conserved molecular systems, the biological consequences of disrupting individual components can vary. This experience encouraged me to approach genetic studies with more openness to complexity and alternative hypotheses.

**If you were able to give one piece of advice to your younger self, what would that be?**

If I could give one piece of advice to my younger self, it would be to start learning programming earlier. Computational skills are essential for large-scale transcriptomic analyses such as RNA-seq, and a stronger foundation in programming would have allowed me to perform more flexible, tailored analyses in my research. In this study, for example, I analyzed alternative splicing events, and I often felt that deeper knowledge of programming would have enabled me to better adapt my computational approaches to specific biological questions.

More broadly, I would also encourage myself to explore topics outside of my immediate area of specialization. Like programming, such skills often become necessary later in a research career and learning them only when the need arises can be time-consuming. If something sparks even a slight interest, it's worth investing time to understand it—it may become unexpectedly valuable in the future.
